# Scalable modEls of Community rehAbilitation for Individuals Recovering From COVID:19 reLated illnEss: A Longitudinal Service Evaluation Protocol—“SeaCole Cohort Evaluation”

**DOI:** 10.3389/fpubh.2021.628333

**Published:** 2021-05-13

**Authors:** Benjamin Kelly, Aidan Innes, Marc Holl, Laura Mould, Susan Powell, Danielle Burns, Patrick Doherty, Greg Whyte, James King, Davina Deniszczyc

**Affiliations:** ^1^Quality and Assurance Directorate, Nuffield Health, Epsom, United Kingdom; ^2^Department for Health, Psychology & Community, Manchester Metropolitan University, Manchester, United Kingdom; ^3^School of Clinical & Applied Sciences, Leeds Beckett University, Leeds, United Kingdom; ^4^Department of Health Sciences, York University, York, United Kingdom; ^5^School of Sport & Exercise Sciences, Liverpool John Moores University, Liverpool, United Kingdom; ^6^National Centre for Sports and Exercise Medicine, Loughborough University, Loughborough, United Kingdom

**Keywords:** COVID-19, rehabilitation, exercise, emotional well-being, digital health, NHS, independent sector

## Abstract

**Introduction:** High levels of physical, cognitive, and psychosocial impairments are anticipated for those recovering from the COVID-19. In the UK, ~50% of survivors will require additional rehabilitation. Despite this, there is currently no evidence-based guideline available in England and Wales that addresses the identification, timing and nature of effective interventions to manage the morbidity associated following COVID-19. It is now timely to accelerate the development and evaluation of a rehabilitation service to support patients and healthcare services. Nuffield Health have responded by configuring a scalable rehabilitation pathway addressing the immediate requirements for those recovering from COVID-19 in the community.

**Methods and Analysis:** This long-term evaluation will examine the effectiveness of a 12-week community rehabilitation programme for COVID-19 patients who have been discharged following in-patient treatment. Consisting of two distinct 6-week phases; Phase 1 is an entirely remote service, delivered via digital applications. Phase 2 sees the same patients transition into a gym-based setting for supervised group-based rehabilitation. Trained rehabilitation specialists will coach patients across areas such as goal setting, exercise prescription, symptom management and emotional well-being. Outcomes will be collected at 0, 6, and 12 weeks and at 6- and 12-months. Primary outcome measures will assess changes in health-related quality of life (HR-QOL) and COVID-19 symptoms using EuroQol Five Dimension Five Level Version (EQ-5D-5L) and Dyspnea-12, respectively. Secondary outcome measures of the Duke Activity Status Questionnaire (DASI), 30 s sit to stand test, General Anxiety Disorder-7 (GAD-7), Patient Health Questionnaire-9 (PHQ-9), Patient Experience Questionnaire (PEQ) and Quality Adjusted Life Years (QALY) will allow for the evaluation of outcomes, mediators and moderators of outcome, and cost-effectiveness of treatment.

**Discussion:** This evaluation will investigate the immediate and long-term impact, as well as the cost effectiveness of a blended rehabilitation programme for COVID-19 survivors. This evaluation will provide a founding contribution to the literature, evaluating one of the first programmes of this type in the UK. The evaluation has international relevance, with the potential to show how a new model of service provision can support health services in the wake of COVID-19.

**Trial Registration:** Current Trials ISRCTN ISRCTN14707226

Web: http://www.isrctn.com/ISRCTN14707226

## Introduction

### Clinical Impact of COVID-19

In late 2019 a highly pathogenic novel coronavirus (CoV), severe acute respiratory syndrome (SARS)-CoV-2, emerged, causing a global pandemic with millions of cases worldwide (1). SARS-CoV-2 commonly attacks the respiratory system, leading to hospitalisation with many requiring breathing support advancing in some cases to intensive care support (2). Further complications include those meeting diagnostic criteria for acute respiratory distress syndrome (ARDS), anaemia, cardiac injury and secondary infection (2). COVID-19 is a highly infectious respiratory disease and as a result the COVID-19 pandemic has profoundly impacted the UK population resulting in strict measures to curtail the spread of infection. This disease was unknown in humans and most research has concentrated on the acute phase to reduce mortality. Acute treatment is largely symptomatic and supportive depending on the severity of infection. As of June 2020, there was no specific treatment or vaccination available. Indications show that COVID-19 will have a profound long-term impact on those infected as was previously seen following the MERS and SARS pandemics. MERS survivors showed significantly negatively impacted HR-QoL for up to 14 months post-virus (3) indicating that rehabilitation should be measured in months/years rather than weeks (4).

Data from previous pandemics such as those described, indicates a number of adverse side effects in recovering patients. Long-term ventilatory dysfunction and associated lung damage are common characteristic as are muscle weakness and fatigue (5, 6). Whilst less common, metabolic disorders, including hyperinsulinemia, insulin resistance, hyperglycemia, and type 1 or 2 diabetes were reported in recovering SARS patients (4).

Non-physical morbidity such as psychological morbidity and cognitive dysfunction are also common after a period of critical illness such as COVID-19. It has been reported that 1 in 10 critically ill patients develop severe psychological problems including anxiety, depression and post-traumatic stress disorder (PTSD) (7). Whilst there are a multitude on contributory mechanisms, the potential areas of comorbidity here all represent important target areas within rehabilitation.

It is anticipated that COVID-19 will hospitalise ~150,000 people by the end of 2020. Of those individuals, it is suggested that 50% will require additional rehabilitation support in the community to support improvement in HR-QoL (8) and to reduce burden on NHS services.

### Impact on Rehabilitation Services

The COVID-19 pandemic has acted as a sharp reminder as to the exceptional work of the National Health Service (NHS). As we move further through the pandemic patients are being medically discharged in growing numbers. As patients move out of the acute phase of care it is clear to see the impending burden facing rehabilitation services, described by the Chartered Society of Physiotherapy as an “tsunami of rehabilitation need” (8). Normal health and social care delivery in many countries, including the UK has been deferred in order to support the acute phase of COVID-19. Healthcare interventions aimed at improving or maintaining function such as falls prevention programmes, as well as well-established rehabilitation pathways such as cardiac and pulmonary rehabilitation, are unable to continue, with potential deleterious effects on function. These issues risk worsening health, physical and psychological function for vast numbers of people who may not have suffered from COVID-19 directly (9). As movement restrictions are lifted, the consequences of these indirect effects of the pandemic will become apparent. Prior to the COVID-19 pandemic, to meet the 18-week standard for newly referred patients and clear the backlog of patients who will have already waited longer than 18 weeks, the NHS would have needed to treat an additional 500,000 patients a year for the next 4 years. The pandemic is likely to make waiting lists grow further and the challenge will be even greater (10).

Alarmingly and as has been made clear by National Institute for Health and Care Excellence (NICE) there is currently no evidence-based guideline available in England and Wales that addresses the identification, timing and nature of effective interventions to manage the physical and non-physical morbidity associated following COVID-19 (11). Progress is being made, with an initial framework devised for assessing early rehabilitation needs of COVID-19 patients, following intensive care treatment. Much more work is required to address the spectrum of needs, particularly for those that have not spent time in intensive care (12). Pulmonary rehabilitation has been shown to be successful in improving exercise tolerance and HR-QoL, and has been shown to reduce hospital admissions rates in patients with COPD (13, 14), yet despite the associated severe muscle wastage and deconditioning, on-going dyspnoea, sleep disorders and severe fatigue, memory problems, anxiety, depression, and post-traumatic stress disorder (15), rehabilitation is neither defined or guaranteed for those recovering from COVID-19.

### New Models of Rehabilitation

A rapid expansion in rehabilitation services is necessary to support an increasing number of patients suffering from long-term complications of COVID-19. Given the level of urgency, a more diverse rehabilitation workforce is required to meet the scale of this challenge, using capacity and skills from sectors outside healthcare organisations. Specifically, improved capacity could be achieved by developing rehabilitation capabilities across the wider non-registered health care staff, including specialist trained exercise professionals, to help meet both demand and effective dose and progression of exercise (16).

The Australian healthcare system may provide a strong basis upon which to base a new model of rehabilitation support, utilising the expertise of exercise professionals. Inclusion of exercise professionals within the Australian healthcare sector has resulted in substantial healthcare cost savings with annual well-being gains of $7,967 and $11,847 per person with diabetes and cardiovascular disease, respectively, with a benefit-cost ratio of 9:1 and 6:1 (17).

Compelling data also exist for the cost effectiveness of exercise in the treatment of mental health, dementia and other common chronic diseases (17) The utilisation of exercise professionals to support clinical rehabilitation is something that has been long employed by Nuffield Health, rendering the Charity well-placed to mobilise and investigate the approach with a cohort of COVID-19 survivors. It should be made clear, that at present there is no formal accreditation pathway for clinical exercise physiologists.

Not only is the organisation of personnel key to new models of rehabilitation, but also the mode of delivery. Delivery modality of rehabilitation will be one of the most significant changes as we progress through the Post-COVID-19 phase. To reduce the number of “face to face” consultations and indeed resource strain, remote consultations including telephone and video platforms have evolved significantly to provide a continuity of care (18). Whilst previous uptake of digital rehabilitation options has been poor (19) this delivery approach has been shown to confer positive health and well-being outcomes in participants that showed strong adherence (19–21). As an example, “Activate Your Heart” is a well-established digital cardiac rehabilitation programme and was evaluated in several different locations. Data demonstrated that users' exercise capacity and HR-QoL improved after completing the programme (22).

The restrictive conditions associated with the COVID-19 lockdown and indeed social anxieties as restrictions are lifted, suggest that there will likely be a greater acceptance of digital healthcare from both a patient and clinician perspective (23, 24). Nuffield health has experience in delivering remote digital interventions for mental health, primary care and physiotherapy. The learnings from these areas will be built into the development of the remote COVID-19 rehabilitation programme. The digital component will be evaluated for both clinical effectiveness as well as acceptability from both the clinician and patient perspective.

### Aims

The aim of the present evaluation is to implement and appraise a novel model of community rehabilitation for individuals recovering from COVID-19. The focus will be on the clinical effectiveness of the programme for improvements in HR-QoL and suppression of COVID-19 related symptoms. The specific research questions include the following:

- Is HR-QoL improved and are symptoms related to COVID-19 reduced at 6 and 12 weeks post-intervention and are benefits retained at 6 and 12 months?
- Is a novel blended model (digital and physical) of care cost-effective in the rehabilitation of those recovering from COVID-19?
- Is a novel blended rehabilitation programme acceptable to both patients and rehabilitation specialists?

We hypothesise that: (a) the 12-week rehabilitation programme will be effective in improving HR-QoL and reducing symptoms related to COVID-19 at 6 and 12 weeks and those benefits will be retained at 6 and 12 months; (b) the blended model will be cost effective when compared to previously described rehabilitation methodologies, specifically outpatient multidisciplinary pulmonary rehabilitation; (c) we expect the programme to be acceptable to both patients and specialists.

## Methods and Analysis

### Trial Design

The protocol presented herein reflects Protocol Number 01. Any amends to this protocol will be detailed in full within the ISRCTN registry. This observational cohort study will be conducted following the STROBE statement (25) for observational studies with the protocol reported in line with SPIRIT Statement (26). We will examine the effectiveness of a 12-week blended community rehabilitation programme on improvements in HR-QoL and reductions in symptoms of COVID-19, in individuals recovering from the disease.

This evaluation will be conducted in concert with the NHS. Initially the programme will be deployed across 4 NHS locations, namely; University Hospitals of North Midlands Trust, Newcastle upon Tyne Hospitals NHS Foundation Trust; Birmingham and Solihull Community Care Group and Central Manchester University Hospitals Trust. Whilst these locations are clustered in the North and Midlands they are demographically and economically diverse. The NHS sites will be serviced by 8 surrounding Nuffield Health Fitness and Well-being Centres, all of which are registered with the Care Quality Commission and are located within a 20-mile radius of a participating NHS site. Each trust will be assigned 2 rehabilitation specialists from Nuffield Health to support the programme. As this intervention will be offered as a National service, recruitment is open ended beginning in September 2020. Nuffield Health intend to expand provision by operationalising all 112 of its fitness and well-being centres in 2021. These locations cover all 7 geographical regions as defined by NHS criteria. An initial evaluation cohort will not exceed 160 participants ensuring that a participant practitioner ratio of 1:10 is not exceeded.

Those wishing to access the programme can do so via NHS referral, this can be through doctor, nurse or other allied health professional such as a physiotherapist. Patients will only be referred if they meet the qualifying criteria presented in Table 1.

**Table 1:** Inclusion and exclusion criteria.

**Inclusion criteria**	**Exclusion criteria**
Previous diagnosis of COVID-19	Active COVID-19 symptoms
Able to walk independently for a minimum of 20 m	Are already receiving community rehabilitation
Must have access to the internet and smartphone/tablet/personal computer (with adequate technological literacy)	Have un-managed medical conditions that contraindicate unsupervised exercise
18 years of age and over	Have a formal diagnosis of post-traumatic stress syndrome, clinically significant anxiety or depression where low intensity mental health intervention will not assist
Access to transport for phase 2 attendance	Have been diagnosed with Chronic Fatigue Syndrome

**Figure 1:**
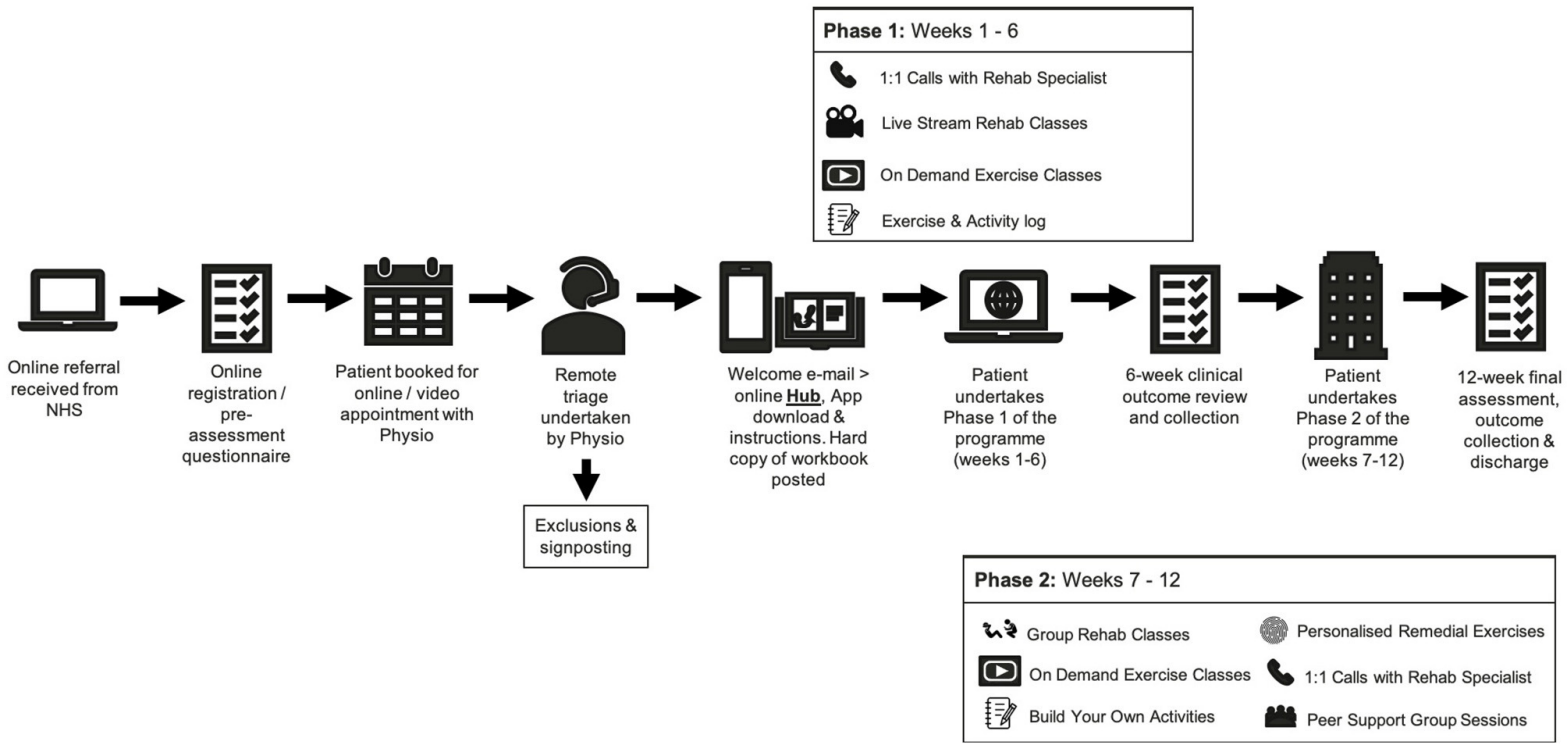
Pathway illustration of the 12-week community rehabilitation programme.

Once referred, the patient will complete an online pre-assessment health questionnaire. Once completed the questionnaire is made available digitally to a specialist trained physiotherapist who will contact the patient to conduct a telephone triage assessment. Following successful triage, the patient is handed on to a rehabilitation specialist who then takes up responsibility of the patients care. All patients will receive the identical 12-week programme structure consisting of two 6-week phases, depicted in Figure 1 and described in detail below.

### Rehabilitation Programme

1. NHS healthcare professionals will utilise inclusion/exclusion criteria at the point of discharge to refer a patient to the programme. Alongside the provision of a patient information document, the patient will be fully informed verbally about the programme, being given the opportunity to join the programme should they so wish. The patient will be made aware that their data will be utilised anonymously for research purposes. The patient may also request that their data is not utilised and will still be able to participate in the programme. Should they choose not to progress they will be sign-posted to alternative community/NHS services where available. If the patient accepts to progress on to the programme the NHS healthcare professional will complete an online referral, sent directly to Nuffield Health using a secure online form. Data sharing agreements have been completed between NHS and Nuffield Health and all processes conform to GDPR and NHS digital requirements.
2. When an online referral is completed, an automated booking process is triggered. Via email or telephone (based on patient preference) the patient will choose an appointment time for an initial triage screening. The patient will also be asked to complete pre-screening questions, designed to support the triage process.
3. The patient next joins a telephone or online video triage consultation utilising this feature. The triage is conducted by specialist physiotherapists trained in remote consultation. The triage is designed to be an additional safety step ensuring that the patient is clinically fit to progress onto the 12-week programme. The 45-min triage will also act to collect additional relevant patient information that may be pertinent when tailoring their exercise programme. Information such as details on additional co-morbidities, emotional well-being and medication will be discussed. Should any contraindications to exercise be identified during the triage the patient will be informed that they are unable to progress on the programme at that time. The patient will be sign-posted back to their General Practitioner, who will also be notified in writing. The original referring clinician will also be notified. At the end of triage, if deemed appropriate, the specialist physiotherapist will refer the patient to the rehabilitation specialist with recommendations for the intensity of entry level exercise and specific needs and goals.
4. Following successful triage, the patient will be automatically sent a welcome pack via post as well as email. This will provide full guidance on how to download, access and register on the digital platforms and will provide links to learning materials. The patients GP will also be made aware that their patient has initiated the programme. The digital application platform utilises the functionality of a platform already used extensively across Nuffield Health (MyTherapy, Nuffield Health, London, UK). All other virtual audio-visual communication will be delivered by a separate digital system (Microsoft Teams, Microsoft, Redmond, USA).
5. Within 72 h of referral, the patient will be contacted by their assigned rehabilitation specialist based at a Nuffield Health site within a 20-mile proximity to the referring hospital. practitioner will provide a welcome to the programme, offer the opportunity to ask any questions and to inform them of the start date of the programme.
6. The patient begins the 12-week programme. This programme phases are as follows:

### Phase 1

Weeks 1–6 will consist of 3 exercise sessions per week. Session 1 will be an online live streamed activity conducted by 2 Nuffield Health trained rehabilitation specialists. One practitioner will run the exercise session and the second will answer questions via the online chat function. The stream will be a 1-way stream, meaning that whilst multiple patients will access the stream at any given time, they will not be recorded/filmed nor will their personal details be visible to the group. A maximum of 10 patients will join a stream at any time. The live stream will last up to 45-min followed by a 15-min period for questions via the secure online chat function or alternatively spoken questions can be provided should the patient have microphone functionality.

The second session of the week during phase 1 will be self-directed. The patient will be directed to a pre-recorded guided exercise session located on a dedicated online platform (Vimeo, New York, USA). This will be a 45-min activity which the patient completes at their leisure. All exercises are designed such that they can be carried out with ease at home.

The third exercise session of the week is described as “build your own.” The patient's workbook provides the patient a menu of activities suitable for them, which they may select to populate an exercise session commensurate with their personal threshold.

Each week the patient will be provided a phone call that will last up to 45-min with the rehabilitation specialist. The aim of the phone call is to listen to any patient queries but to also offer support on themes such exercise selection, symptom management and emotional well-being. Nuffield Health specialists have comprehensive training in each of these areas. Prior to progressing to Phase 2 patients will receive a remote mid-point review by the rehabilitation specialist. Progress will be discussed in detail and the patient will be asked if they feel ready and willing to progress to phase 2. If their progress is deemed insufficient, the patient will be recommended to complete another 6-week digital programme in full, before moving into a gym-based setting. Progress will be reviewed weekly and patients will be able to join the face to face component at a later point. All group-based sessions will be offered at two time points across the course of the day, with an AM and PM option. All one to one activity such as the weekly phone calls will be booked according to participant preference on a weekly basis. All above processes intended to maximise participant retention throughout the course of the evaluation.

### Phase 2

Following successful completion of phase 1, patients will progress to the phase 2 face to face programme. This phase will be conducted in strictly controlled gym environments conforming to all necessary Government and Public Health England (PHE) guidance. As per phase 1, phase 2 will consist of 3 exercise sessions per week. The first session of the week will be a rehabilitation specialist lead group exercise programme. In appropriately prepared and ventilated spaces, groups of up to 5–10 patients will engage in a 45-min exercise class followed by 15 min for questions and answers. Exercises will consist of aerobic and strength-based exercises as well as stability and mobility. In order to promote continued self-management, the second exercise session of the week will be a remote pre-recorded session that the patient will carry out at home independently, as per phase 1. Similarly, the third session of the week, “build your own” will remain; however, the patient will be encouraged to complete this session within a supervised gym environment. The rehabilitation specialist will be on hand within pre-defined time slots to provide advice and guidance. The patient will again receive a weekly consultation with the rehabilitation specialist following the aforementioned themes. This will culminate between weeks 12 and 13 with a final assessment, summary report and sign-posting to additional services where required.

### Practitioner Recruitment and Training

Rehabilitation specialists will be recruited and trained from a pool of exercise professionals working within Nuffield Health. All exercise professionals have a foundation training to a minimum of The Level 3 Personal Training Qualification from an accredited training provider, with preference for professionals trained to level 4. The competencies associated with these qualifications can be found elsewhere. All professionals are registered with The Chartered Institute for the Management of Sport and Physical Activity (CIMSPA). As part of this registration, all professionals are required to engage in continued professional development (CPD) as part of their contract of employment, with a pre-requisite to attain a minimum of 10 CPD points each year. Given the unique structure of Nuffield Health, all exercise professionals have experience working with clinical populations and work closely with clinical professionals on a daily basis within a shared learning environment.

Utilising the Nuffield Health accredited training academy, a multi-disciplinary team of clinical and exercise experts as well as experienced clinical operations specialists will deliver a comprehensive programme of training to up skill exercise professionals to COVID-19 rehabilitation specialists. At present no external standards exist regarding specific competencies in this area. The design of the programme and its content has however been carried out in collaboration with NHS representatives and key authorities from organisations leading rehabilitation nationally.

Training will be delivered via a blended learning approach utilising a mixture of interactive virtual classrooms, online learning, webinars and question and answer sessions. The content to be included covers:

- An overview of the clinical impact of COVID-19, long term effects and the requirement for community rehabilitation and its goals.
- Roles and responsibilities of those involved in delivery of the rehabilitation programme.
- Physiotherapists will be refreshed on initial subjective assessment, screening for inclusion/exclusion and need for onward referral, use of outcome measures and handover process to ensure seamless patient journey.
- Exercise professionals cover week by week roles and responsibilities, systems training, outcome measures, red flags and escalation processes.
- Exercise professionals will further refresh and advance coaching skills, exercise programming, great conversations skills, exercise progression, and regression.
- All will receive mental Health First Aid training—recognising signs and symptoms of emotional distress and understanding how to signpost to appropriate treatment pathway.

All training will be assessed via formal assessment testing theoretical knowledge via online examination and practical skills assessed via role play scenarios and “course-work” tasks.

### Patient and Public Involvement

Patients were first involved in this evaluation at their point of clinical referral following a 12-week post discharge follow up. The research questions posed within the protocol paper were constructed with the support of NHS clinicians whom work directly with this clinical population as well-members of NHS Trust management. We believe that the research questions reflect the immediate and on-going needs of the NHS who have a strong insight as to their patient's needs.

Qualitative feedback will be collected from patients throughout their rehabilitation. An initial cohort of 100 patients will be invited to review each milestone of the programme as part of a focus group following their rehabilitation. An evaluation period will then be employed to refine the pathway based on patient feedback, this will include feedback on outcome and recruitment methods.

A patient survey will be provided to all participants that were eligible for the rehabilitation programme gauging views on the dissemination plan and how the intervention may further integrate into community settings.

## Measures

Outcome data will be collected at 0, 6, and 12 weeks and again at 6- and 12-months post-intervention. Self-report data will be collected via digital application (MyWellbeing, Nuffield Health, London, UK). The patient will be emailed and provided a push-notification prompting them to complete the aforementioned questionnaires. Whilst this evaluation will not utilise any formal comparison group, it is intended that collaboration with trusts will support the analysis of anonymised “usual care” outcome data. This is likely to come from community physiotherapy and/or modified pulmonary rehabilitation.

All data will be securely stored on a dedicated Nuffield Health server and will be retained in line with the organisations publicly available data retention schedule. Modifications to data written to the database will be documented through via an internal inquiry system. Data entered into the database will be retrievable for viewing throughout by those granted secure access privileges associated with an identification code and password. Any data errors will be summarised along with detailed descriptions for each specific problem in a data query reports, which will be sent to the study Outcomes Analyst. The Outcomes Analyst will check any inconsistency, checking other sources to determine required corrections. Any coding changes required within the digital data capture system will then be implemented within 24 h.

Complete back up of the primary database will be performed twice a month. Incremental data back-ups will be performed on a daily basis. In addition to system back-ups, additional measures will be taken to back-up and export the database on a regular basis at site level. The outcomes analyst will send weekly email reports with information on missing data, missing forms, and missing visits to site level co-ordinators who will then rectify immediately as and when required. Data security audits will be completed by the Nuffield Health Information Security group on a quarterly basis. Full details of group membership and details of audit processes can be provided upon request.

### Primary Outcome Measures

#### EuroQoL Five Dimension Five Level Version (EQ-5D-5 L)

This measure is used to assess a person's perception of their general health state and obtain a measure of quality adjusted life years (QALYs). Outcomes can be benchmarked against UK population norms. It covers five dimensions: mobility, self-care, usual activity, pain/discomfort and anxiety/depression, which are rated by the person on five levels of severity: no problems, slight problems, moderate problems, severe problems and extreme problems/unable to function within that domain (27).

### Dyspnea-12

Dyspnea-12 consists of 12 descriptor items on a scale of none (0), mild (1), moderate (2), or severe (3). It provides an overall score for breathlessness severity that incorporates seven physical items and five affective items. The time reference period for “these days” captures the current level of breathlessness experienced by patients as opposed to specifically on the day of the test or in response to a specific activity. Total scores range from 0 to 36, with higher scores corresponding to greater severity (28).

### Secondary Outcome Measures

#### Duke Activity Status Index

The Duke Activity Status Index (DASI) is an assessment tool used to evaluate the functional capacity of patients with cardio-pulmonary diseases (29). The activities in the scale are chosen to represent major aspects of physical function, i.e., personal care, ambulation, household tasks, sexual function, and recreational activities. As such, these responses can also be used to assess physical limitations relevant to the patient's HR-QoL. Responses from 12 items are summed up to get a total score, which ranges from 0 to 58.2. Higher scores indicate a higher functional capacity.

### 30 s Sit to Stand Test

The 30 s Sit to Stand Test is utilised for testing strength and endurance in a variety of cohorts. It is part of the Fullerton Functional Fitness Test Battery (30). This test was developed to overcome the floor effect of the 5 or 10 repetition sit to stand test in older adults. The 30-s chair stand involves recording the number of stands a person can complete in 30 s rather than the amount of time it takes to complete a pre-determined number of repetitions. That way, it is possible to assess a wide variety of ability levels with scores ranging from 0 for those who cannot complete 1 stand to > 20 for more fit individuals.

### Generalised Anxiety Disorder-7

GAD-7 comprises 7 items measuring symptoms and severity of anxiety based on the DSM-IV diagnostic criteria for GAD. The GAD-7 has good internal consistency (α = 0.92) and good convergent validity with other anxiety scales. Higher scores indicate greater severity of symptoms. The GAD-7 has increasingly been used in large-scale studies as a generic measure of change in anxiety symptomatology, using a cut-off score of 8 (31).

### Patient Health Questionnaire-9

The PHQ-9 is a self-report measure of depression that has been widely used in research and is a regular screening measure utilised in primary care and hospital settings. The PHQ-9 items reflect the diagnostic criteria for depression outlined by the Diagnostic and Statistical Manual of Mental Disorders, Fourth Edition—Text Revision (DSM–IV–TR) (32). Summary scores range from 0 to 27, where larger scores reflect a greater severity of depressive symptoms. The PHQ-9 has been found to discriminate well between depressed and non-depressed individuals using the cut-off total score ≥10, with good sensitivity (88.0%), specificity (88.0%) and reliability (33).

### Patient Experience and Programme Acceptability

The Patient Experience Questionnaire (PEQ) instrument will be used to assess patient experience and satisfaction. The PEQ contains several quantitative questions and open-ended questions that are used to assess participant's views and satisfaction with service provision (34).

### Costs

EQ-5D-5 L utilities will be reported alongside the full evaluation costs of the intervention so as to elicit a “per head” economic evaluation of all participants recruited (35).

### Engagement and Usage Measures

The digital systems will collect anonymized descriptive data relating to engagement and usage of the service users with the platforms. Data collected will include the number of sessions attended, time spent in the platform, number of activities completed, number of minutes per log-in, number of resources accessed. A session is defined as an instance where a user logs on to the system. Session time will be always an imperfect calculation, as users may take breaks within a session, without formally log out of the system. To prevent this overestimation, periods of more than 30 min without interaction will be taken as 1 min and periods of inactivity longer than 3 h will start the count on a new session. Use of different program components will be measured.

### Statistical Analysis

Participation levels will be monitored throughout the programme and reasons for withdrawal or non-compliance will be recorded. All participants are selected according to clinical criteria alone with no other factors influencing participation so as to limit selection bias. Information biases are limited via the prospective nature of this evaluation and the methodology employed to collect mandatory data at each designated time point. As well as the primary and secondary outcome measures being collected, a detailed clinical history and additional triage will be undertaken for each participant. This will limit confusion bias through the identification of relevant confounding clinical variables. Efficacy of treatments over time will be measured using mixed effects models. To complement the *post-hoc* comparisons, the magnitude of change on the primary and secondary outcomes measures will be established using Cohen's *d* statistic. Bonferroni corrected *p*-values will be reported for multiple comparisons. Participants with missing data will be removed for analysis with complete-case analysis being utilised.

An interim-analysis will be performed on the primary endpoint when 50% of participants have completed up to the 6-month follow up point. The interim analysis will be performed by an independent statistician. The statistician will report to the principal investigator (BMK) only. The principal investigator will have unblinded access to all data and will discuss the results of the interim analysis with the project team.

### Ethics and Dissemination

Manchester Metropolitan University Ethics Committee approved this study on 29/09/2020 (Ref: 25307). Informed consent will be gained from all participants prior to referral onto the rehabilitation programme. Consent will be captured digitally as part of the online referral programme.

Participants will be contacted weekly to ensure that any clinical concerns are addressed and escalated where relevant. Processes are in place to inform the referring clinician and the participants general practitioner should medical intervention be required.

The principal investigators will have access to the cleaned data sets. Project data sets will be housed securely within the project database hosted on a secure Nuffield Health server. Should data sharing be required under reasonable request (e.g., with the NHS) a secure file transfer protocol will be created for the study, and all data sets will be password protected. To ensure confidentiality, data dispersed to project team members will be blinded of any identifying participant information.

Pilot data is expected by December 2021 and will be published in an open access journal. Any intellectual property pertaining to successful delivery of the service will be shared directly with NHS partners.

## Discussion

New models of rehabilitation are urgently required to address the immediate gap in provision for those recovering from COVID-19 as well as the escalating back log of rehabilitation cases nationally. Nuffield Health, the UK's largest not-for-profit healthcare charity, have long prioritised exercise as a first line intervention for the treatment and prevention of long-term conditions. This has been successfully achieved via a uniquely structured estate linking hospitals and health and well-being centres as well as the up skilling of exercise professionals to work with clinical populations. Now, by working closely with the NHS, a unique learning partnership will assist in the development of a new rehabilitation pathway, that may later evolve to utilise the expertise of the fitness sector in supporting the NHS and its rehabilitation needs. We must also later review in detail how we create a model that has utility beyond the healthcare system within the United Kingdom, such that patients are able to benefit from this level of support internationally, within varying healthcare structures.

Undertaking the principal aim of this trial will allow for a robust test of the effectiveness of a new 12-week blended rehabilitation programme within a population that has not previously been investigated within a community rehabilitation context. Specifically, this work will provide new insight into changes in HR-QoL and disease specific symptoms related to COVID-19 following 12-weeks of exercise rehabilitation. Positive results of these main outcome measures will allow the programme to consolidate itself as not only a valid treatment option, but as an essential component to the care management pathway of COVID-19 survivors, and indeed those recovering from other serious conditions.

The novel structure of the programme will support further expansion of digital components within the rehabilitation of those recovering from serious illness. This is relevant not just for improving access to information and efficiency of data collection but critically, the remote delivery of care and the ability to individualise programmes of rehabilitation. The relevance of the results will likely have implications for the implementation and success of blended rehabilitation models i.e., digital and physical combined, across health care systems worldwide.

Examining potential mediators and moderators of change will contribute to our understanding of key processes in achieving improvement in services using blended models of rehabilitation. Whilst mediators and moderators of rehabilitation outcomes have been explored, this will be the first exploration of a combined digital/physical intervention. This will inform the tailoring of interventions to best address the needs of the targeted population, ultimately leading to the development of more effective interventions.

An area requiring on-going review and indeed development relates to the provision and impact of rehabilitation across sociodemographic groups. Black, low-income, and immigrant communities are particularly vulnerable and disproportionately impacted by COVID-19 (36, 37). Furthermore, data exists indicating that secondary care-based clinics may be underused by older populations and those in poorer socioeconomic circumstances (37). The proposed evaluation will ensure that sociodemographic variation is considered within analysis and that a representative sample from those that are disproportionately affected are consulted post-programme to understand barriers and facilitators. It is critical that learnings are continuous and that they are built into future re-iterations of the rehabilitation programme.

The proposed economic analysis will add to the current literature in regard to evidence of the cost-effectiveness of home or web-based rehabilitation programmes and will be an innovative analysis of a clinical rehabilitation programme ran independently of allied health professionals within a non-NHS community environment. In a context of health care provision where resources are now especially stretched, cost-effective interventions can only support the delivery of effective health services.

In summary, COVID-19 has proven devastating in its cost of life and long-term impact on survivors. Scalable interventions must be developed to address what will be a long-term requirement in the rehabilitation of patients having suffered critical illness. To achieve rapid scalability, blended interventions will soon become a recognised viable option across health care. They can be beneficial both in costs and resource management within the NHS and will bring disparate sectors closer together in a combined mission of improving the health of the nation. It is critical that as technology rapidly develops, supporting innovative models of care so too must research in order to rapidly continually and rapidly update on the benefits of providing blended community rehabilitation. This long-term evaluation aspires to drive research and innovation forward, and in doing so support the NHS in its aim of controlling the impacts of COVID-19 and delivering on its long-term plan.

## Summary

### Strengths and Limitations of This Study

- Evaluates a critical and novel patient cohort.
- This evaluation will review the impact of digital and physical approaches to rehabilitation.
- Evaluates a new model of care delivery and the training of non-clinical staff.
- Demonstrates strong example of NHS/independent sector collaboration.
- A significant proportion of data will be self-reported due to COVID-19 restrictions.

## Ethics Statement

Manchester Metropolitan University Ethics Committee approved this study on 29/09/2020 (Ref: 25307). Informed consent will be gained from all participants prior to referral onto the rehabilitation programme. Consent will be captured digitally as part of the online referral programme. In review Participants will be contacted weekly to ensure that any clinical concerns are addressed and escalated where relevant. Processes are in place to inform the referring clinician and the participants general practitioner should medical intervention be required.

## Author Contributions

BK, AI, and PD conceived and designed the original protocol. BK, AI, PD, MH, LM, DD, DB, SP, and GW were involved in amending the protocol. BK coordinated the programme throughout and wrote the initial draft of the manuscript with contributions from all authors. All authors further contributed to subsequent drafts and have read and approved the manuscript.

## Conflict of Interest

JK reports no conflict of interest however is a voluntary member of Nuffield Health's External Research Advisory Board. BK, AI, MH, LM, and DD report personal fees from Nuffield Health outside of the submitted work. SP was a member of Nuffield Health's External Research Advisory Board and holds a Chair of Public Health position within Manchester Metropolitan University, funded by Nuffield Health. The development of this pathway has been funded by Nuffield Health (Registered Charity Numbers: 205533 in England and Wales and SC041793 in Scotland). The remaining authors declare that the research was conducted in the absence of any commercial or financial relationships that could be construed as a potential conflict of interest.

## Abbreviations

ARDS: Acute respiratory distress syndrome
AUC: Area under the curve
CIMSPA: The Chartered Institute for the Management of Sport and Physical Activity
CK: Creatine kinase
COVID-19: Corona virus disease
CPD: Continued professional development
EQ-5D-5L: EuroQoL Five Dimension Five Level Version
GAD-7: General anxiety disorder assessment-7
H7N9: Avian influenza
HR-QoL: Health Related Quality of Life
ICU: Intensive care unit
IL-6: Interleukin-6
MERS: Middle East respiratory syndrome
NHS: National Health Service
PEQ: Patient experience questionnaire
PHQ-9: Patient health questionnaire-9
PTSD: Post-traumatic stress syndrome
QALY: Quality adjusted life years
SARS: Severe acute respiratory syndrome.
